# Simulating language knowledge across the EU: language regimes, language learning and consequences for linguistic disenfranchisement

**DOI:** 10.1007/s42001-021-00137-5

**Published:** 2021-08-25

**Authors:** Marco Civico

**Affiliations:** Université de Genève, Geneva, Switzerland

**Keywords:** Languages, Europe, Linguistic disenfranchisement, Simulation

## Abstract

The objective of this paper is to develop an simulation model able to test different language education orientations and their consequences for the EU population in terms of linguistic disenfranchisement, that is, the inability of citizens to understand EU documents and parliamentary discussions should their native language(s) no longer be official. I will focus on the impact of linguistic distance and language learning. Ideally, this model would be a tool to help EU policy makers make informed decisions about language practices and education policies, taking into account their consequences in terms of diversity and linguistic disenfranchisement. The model can be used to force agents to make certain choices in terms of language skills acquisition. The user can then go on to compare different scenarios in which language skills are acquired according to different rationales. The idea is that, by forcing agents to adopt certain language learning strategies, the model user can simulate policies promoting the acquisition of language skills and get an idea of their impact. In this way, the model allows not only to sketch various scenarios of the evolution of language skills among EU citizens, but also to estimate the level of disenfranchisement in each of these scenarios.

## Introduction

Though to a lesser extent than other parts of the world, such as Africa, Europe is traditionally a culturally and linguistically diverse continent. Throughout the history of Europe, diversity has often represented a major obstacle to peaceful cohabitation. Multilingualism is but one of the numerous aspects of European diversity. Therefore, it is not striking that more than half a century since the beginning of the big European integration project known today as the European Union, linguistic diversity is still a hotly debated issue. Discussions on EU multilingualism revolve around some recurring macro-issues, such as integration [23], education [25], and trade [15]. Many of these discussions have important ideological connotations, which often boil down to one simple question: should EU multilingualism be supported, neglected or even discouraged by the institutions?

Multilingualism is also a hot topic when it comes to discussions on the administration and functioning of the Union [19]. As a consequence of the enlargement of the EU since the early 2000s, the number of languages has increased dramatically. It is not unusual to read in the general press complaints about a presumed perception among EU citizens of the EU language services (generally divided between translation and interpreting) as a costly and cumbersome apparatus. The most common reply to this comment is that giving up language services would put at risk the capability of every EU citizen to fully understand what is going on in the EU. Besides, one should keep in mind that the cost of language services is small compared to other possibilities, such as making every EU citizen fluent in at least one *lingua franca* (often assumed to be English) to the point of being able to participate fully in the political life, should they wish to. This argument is further reinforced by the fact that this would not only represent an increase in the overall expense for multilingualism, but also a transfer of the financial burden from the EU institutions to the individual member states or even to individual citizens. Besides, multilingual skills are not evenly distributed across demographic groups. Therefore, giving up language services would unfairly put some specific categories at a disadvantage (for example, elderly people, people with low income and, clearly, non-native speakers of the chosen *lingua franca*) [17].

A language regime is roughly defined as the set of rules and measures concerning the language(s) “permitting complete mutual comprehension in a ‘deliberation’ among representatives of language groups” [27, p. 164]. As of today, all EU institutions adopt a full language regime where the official languages of the member states are also official languages of the EU, at least when it comes to communication between the institutions and the outside.^1^ Some institutions, though, may have a different arrangement for inter- and intra-institutional communication. Among all EU institutions, the European Parliament seems to be the one that accommodates language diversity the most. Indeed, no specific foreign language skills can and should be expected from elected members of the Parliament, and all EU citizens should be able to participate, actively or passively, in the parliamentary debate, regardless of their linguistic profile. Restricting communication to one or a few selected languages might push some people further away from the institutions, working against the ultimate goal of an integrated Europe.

Many scholars have discussed the consequences of different strategies of approaching multilingualism in the EU [16, 19, 20]. In particular, some have brought forward the idea of “linguistic disenfranchisement”, which is roughly defined as the condition of citizens who would lose their ability to understand EU documents and parliamentary discussions, should the full multilingualism regime be abandoned by the institutions. In this paper, I develop a tool to sketch and project the linguistic landscape of the EU by means of computational simulations. In particular, I test how different strategies in terms of languages to learn lead to different outcomes in terms of overall disenfranchisement of EU citizens. The tool is developed as a simulation model that depicts the distribution of languages across the EU by number of speakers and project the evolution of this distribution based on different language learning strategies. On the basis of these projections, the model shall:

1. evaluate the evolution of language skills among EU citizens, based on different approaches to the choice of the language(s) to learn;
2. estimate the level of linguistic disenfranchisement based on different options of language regime for the EU; and
3. as a further development of point 2, estimate the relative impact of disenfranchisement across different social groups (e.g., what socio-economic or demographic classes are more affected).

Simulation models lend themselves very well to the treatment of language-related matters. In the past, they have been applied particularly to the study of language competition. For example, Minett and Wang [24] are among the first to apply simulations to study the phenomenon of language contact and explore the effect of language status and education policies on the long-term survival of minority languages. Castelló, Loureiro-Porto, and San Miguel [5] provide another example and use simulations to analyze the dynamics of a community where two languages are spoken, each with a share of monolingual and bilingual speakers. More specifically, they study the impact of language status and individual likelihood to shift to speaking another language on minority language growth and decline. For another example, Civico [6] develops a simulation model for the study of the competition between German and Romansh in the Swiss canton of Grisons.^2^

The rest of the paper is structured as follows. In Sects. “Language diversity in Europe” and “EU language regimes and linguistic disenfranchisement”, I discuss multilingualism in Europe and in the European Union, along with its historical evolution. In particular, I explore in more depth the notion of language regime, its legal bases and practical implications within various EU institutions. In Sect. “A model of language acquisition”, I discuss language skills among EU citizens. I present the methodology adopted in this paper to study linguistic diversity and linguistic disenfranchisement. Therefore, I discuss the development of the model and the data used. Finally, in Sects. “Results and discussion” and “Final remarks and future research”, I present the main results of the model and conclude by addressing its main limitations.

## Language diversity in Europe

In this section, I discuss language diversity in Europe, focusing in particular on the territory of the European Union. This section is not an in-depth analysis of the linguistic landscape of Europe, which would be largely beyond the scope of this paper. Rather, it is intended as a brief overview that shall help the reader familiarize herself with linguistic diversity in Europe and to show why it cannot be neglected by the institutions.

As mentioned in the introduction, Europe possesses a significant level of cultural and linguistic diversity. Most languages spoken across Europe belong to the Indo-European language family, with some notable exceptions, such as Finnish, Hungarian, Estonian (Uralic languages), Maltese (Semitic) and Basque (a language isolate). Within the Indo-European language family, Romance, Germanic and Slavic languages are by far the most commonly spoken. Smaller sub-families include Hellenic, Celtic and Baltic languages. If we also take non-indigenous languages into consideration, a number of African and Asian languages are added to the picture, such as Arabic, Turkish and Chinese. According to Ethnologue,^3^ a total of 288 languages are currently spoken by about 741 million people throughout Europe (including the whole Russian Federation and not counting Turkey). The overwhelming majority of these languages are spoken by minorities. It has been observed that linguistic diversity (understood as the collection of living languages) is on a decreasing trend in Europe. Indeed, more than 40% of these 288 languages are classified as either “in trouble” or “dying” according to the EGIDS (Expanded Graded Intergenerational Disruption Scale, a scale that classifies languages based on their level vitality),^4^ meaning that these languages are threatened with extinction within a few generations.

The only Europe-wide treaty on the protection and promotion of regional and minority languages is the European Charter for Regional or Minority Languages (ECRML), adopted by the Council of Europe in 1992 and ratified by a majority of its member states (with the notable exceptions of, among others, France and Italy, which signed the Treaty but have yet to ratify it). The ECRML applies to all languages that are

1. traditionally used within a given territory of a State by nationals of that State who form a group numerically smaller than the rest of the State’s population, and
2. different from the official language(s) of that State,

thereby excluding recent immigrant languages. Generally speaking, languages listed in the Treaty are granted different levels of support, ranging from the mere recognition of such languages as an expression of cultural wealth to the implementation of *ad hoc* education plans.^5^

Migration flows also play a major role in shaping and reshaping Europe’s linguistic landscape. Language and migration are linked by a double thread. On the one hand, people who migrate to Europe bring their language(s) along with them, which then end up being used within communities of migrants with the same linguistic background. On the other hand, a common language (as well as other forms of cultural proximity) can act as a driver for migration. Beine, Docquier, and Rapoport [1] and Grogger and Hanson [21] find that sharing a common language increases migration flows. Belot and Ederveen [2] find that migration flows are positively correlated with the linguistic proximity^6^ between the language of the origin and that of the destination. Interestingly, they find that the impact of linguistic proximity on migration flow is twice as strong as that of per capita GDP of the destination country.

On top of all this, the acquisition of skills in languages other than one’s own is highly encouraged within the European Union with a view of strengthening intercultural comprehension across a highly diverse territory. In a sense, one could argue that this is a way of “acquiring” diversity, especially if one assumes that learning a foreign language does not only mean acquiring language skills, but also being exposed to the culture and traditions that come with it. The EU is officially committed to fostering multilingualism among its citizens, by working together with Member States to reach ambitious objectives, such as “teaching at least two languages in addition to the main language(s) of instruction from an early age and by exploring the potential of innovative approaches to the development of language competences” [8]. The EU recognizes that “[i]ncreasing and improving language learning and teaching could strengthen the European dimension in education and training [and] foster the development of a European identity in all its diversity [...]. Multilingual competence provides a better understanding of other cultures, thus contributing to the development of citizenship and democratic competences” [9].

All in all, one could argue that languages are simultaneously a manifestation and a driver of diversity. As I discuss in the next section, the European Union has always acknowledged the importance of diversity and eventually gave it legal recognition through Article 22 of the Charter of Fundamental Rights of 2000, which states that “the Union shall respect cultural, religious and *linguistic* diversity” (emphasis added) [14, art. 22, p. 13].

## EU language regimes and linguistic disenfranchisement

In this section, I discuss the notion of language regime, its application in the context of the European Union and its implications in terms of linguistic disenfranchisement. Concerning the latter point, I present a number of different language regimes that shall serve as a basis for the simulation model. Besides, I review a few previous studies on this topic.

### Language regimes in the EU

The legal basis of the current multilingual regime of the EU is Regulation No. 1 adopted by the Council on 15 April 1958. The Regulation lists rules identical to those applied by the ECSC. In its original version, it officially provides that “[t]he official languages and the working languages of the institutions of the Community shall be Dutch, French, German and Italian” [7] and it has been amended with every enlargement to include new languages. From a legal standpoint, this means that all texts are drafted in all official languages, they are not mere translations of an original version and are all equally binding.^7^ It is worth noting here that there exists a difference between the *external* and the *internal* language regime. Most of the preceding considerations apply to the external language regime, which covers all exchanges between the institutions and the outside. On the contrary, the internal language regime governs the exchanges within and between the institutions of the Community. Regulation 1/1958 allows for a great deal of flexibility within institutions by providing that “[t]he institutions of the Community may stipulate in their rules of procedure which of the languages are to be used in specific cases” [7, art. 6]. It was observed that French, German and, starting from the 80s, English quickly became the languages of everyday use within the institutions [12]. Some even go as far as to argue that French was the only official language of the European Economic Community up until 1973, when the UK and the Republic of Ireland joined the Community [22]. The European Commission explicitly lists English, French and German as its “procedural” languages for internal business.^8^

At the time that I started developing the model, the European Union had 28 member states and 24 official and working languages (OWLs).^9^ However, for a more complete representation of the linguistic landscape of the European Union, a number of regional and minority languages and dialects would need to be mentioned in addition to the list of official languages. For example, although Scottish Gaelic enjoys equal status (or, in the words of the Gaelic Language Act, equal *respect*) as the English language in Scotland,^10^ it is not one of the official languages of the EU. Although by “regional and minority languages” one usually refers to languages traditionally spoken on the European territory, we should not disregard the weight of non-autochthonous language communities that resulted from migration flows. Some of these languages even have local recognition in some member states. It is the case, for example, of Vietnamese, which is an officially recognized minority language in the Czech Republic. Other immigrant languages do not have official recognition in any EU member states but are still spoken by a sizeable share of people as a native language, such as (different varieties of) Arabic in France [29].

### Types of language regimes

Various authors have discussed different types of language regimes. Often, language regimes vary simply in the number of languages they include, although other variables can come into play. Referring to Pool [26] and Gazzola [16], Grin [20] provides a detailed classification of the different types of language regimes for multilingual international organizations that have been discussed over time. He lists seven types of language regimes:

1. the “monarchic” regime, in which there is only one official and working language (often English, in the case of the European Union), implying that everybody needs to be able to speak the chosen language and that there is no need for language services;
2. the “synarchic” regime, which works in the same way as the monarchic one, with the only difference that the chosen language is not native to the citizens of any member state (such as Esperanto);
3. the “oligarchic” regime, in which there are more than one official and working language (for example three or six), implying a need for translation and interpretation services from and into these languages and that everybody is fluent in at least one of them;
4. the “panarchic” regime, in which all languages are official and working language of the institution (which is currently the case of the European Parliament), implying a need for translation and interpretation services between all these languages, but no need for foreign language skills;
5. the “hegemonic” regime, which works like the panarchic regime, with the only difference that translation and interpretation does not happen directly between all languages, but through a “pivot” language, such as English;
6. the “technocratic” regime, which is the same as the hegemonic one, with the difference that the pivot language is no one’s native language, such as Esperanto;
7. finally, the “multiple symmetrical relay”, which works like the hegemonic regime, but relying on *n* pivot languages instead of one (often with $n=3$, in which case we speak specifically of “triple symmetrical relay”).

Table 1, adapted from Grin [20], summarizes the seven language regimes, assuming that the language chosen for the monarchic and hegemonic regimes is English, the one for the synarchic and technocratic ones is Esperanto, and the ones for the oligarchic one are English, French and German.^11^

**Table 1:** Various types of language regimes (Grin [20])

Regime	Number of OWLs	Nature of OWLs	T&I directions	Foreign language learning needs
Monarchic	1	English	0	English by all non-Anglophones
Synarchic	1	Esperanto	0	Esperanto by all
Oligarchic	$1<n<24$	English, French, German	6	English, French or German by non-native speakers
Panarchic	24	All 24	$n(n-1)=552$	None
Hegemonic	24	All 24	$2(n-1)=46$, via English	None
Technocratic	24	All 24 + Esperanto	$2n=48$, via Esperanto	None
Triple symmetrical relay	24	All 24	$r(2n-r-1)=132$, where $r=3$	None

### Linguistic disenfranchisement

Linguistic disenfranchisement, first introduced by Ginsburgh and Weber [19], is a direct consequence of different language regimes. The rate of linguistic disenfranchisement measures the share of a population that is excluded from the group of beneficiaries of a given language policy [4]. In the specific context of the European Union, the linguistic disenfranchisement rate is generally defined as the percentage of citizens who do not master any official language. These people would be unable to understand EU documents and participate in the EU political discussion, either actively or passively, following a reduction of the number of working languages of EU institutions. Ginsburgh and Weber [19] further distinguish *dichotomous* and *distance-adjusted* (or *continuous*) disenfranchisement rates. The dichotomy of the first dimension refers to the fact that an individual either speaks or does not speak a given language, regardless of anything else. The second dimension adds linguistic distance to the picture. Linguistic distance can be roughly defined as an index (between 0 and 1) that captures the (mostly lexical) similarity between two languages, higher values indicating greater similarity.^12^ An individual is considered disenfranchised if she does not speak any OWL, but the level of disenfranchisement that she experiences is a function of the linguistic distance between her language(s) and the OWL(s). For example, if we imagine a language regime that only has Spanish as an OWL, an individual speaking Italian would be “less disenfranchised” than an individual speaking German, due to Spanish being closer to Italian than it is to German.

Regardless of whether we take on a dichotomous or a distance-adjusted approach, when deciding whether an individual is disenfranchised, one can take into consideration either an individual’s native language only, or all the languages in which she is proficient. The first case only looks at native vs non-native speakers. Therefore, one is considered disenfranchised simply if one’s *native* language is not included in the list of OWLs, regardless of one’s skills in other languages. In the second case, one is counted as disenfranchised if one does not speak any OWL, considering one’s native language and any other language one is able to speak. The four indices can, therefore, be arranged in a two-by-two matrix in which the two dimensions are “dichotomous vs distance-adjusted” and “native vs all languages”. When looking at second languages, one might also want to take into consideration the level of proficiency in said second languages. One might indeed consider as disenfranchised all those who have a less-than-proficient knowledge of a language. This is equivalent to expanding the “native vs all languages” dimension to include any nuance of knowledge between basic and native-like. This distinction is also discussed by Gazzola [18], who speaks of *absolute* disenfranchisement, which considers as disenfranchised all individuals having absolutely no knowledge of the official language(s), and *relative* disenfranchised, which considers as disenfranchised also individuals who have less-than-proficient knowledge of the official language(s). The two disenfranchisement rates are calculated, respectively, as follows [18]:

1 $$\begin{aligned}&D_\alpha =1-S_\mathrm{b} \end{aligned}$$

2 $$\begin{aligned}&D_\mathrm{r}=1-(\mathrm{NS}+\mathrm{NNS}_\mathrm{p}) \end{aligned}$$

where $D_{\alpha }$ and $D_\mathrm{r}$ are, respectively, the absolute and relative disenfranchisement rates, $S_\mathrm{b}$ is the percentage of residents who have *at least some* knowledge of *at least one* official language, *NS* is the percentage of native speakers of the official language(s) and $\mathrm{NNS}_\mathrm{p}$ is the percentage of non-native speakers who are proficient in the official language(s). The indicator used for the analyses in the rest of this paper is the *relative* disenfranchisement rate.

## A model of language acquisition

In this section, I present in detail the model developed for the purposes of this paper. The model is written using the Python programming language. The environment simulated is a collection of agents that represent roughly the linguistic and socio-economic profile of the European Union. As said in the introduction, this model can be used to test different language learning strategies among agents. This can be thought of as the consequence of education policies. For example, one might wonder what the consequences would be if EU member states favored (e.g., through education policy) the acquisition of skills in one of the most spoken languages or in languages more closely related to people’s native language. As I will discuss in greater detail in “Simulating language learning”, the model allows agents to pick a second or third language to learn on the basis of different rules, namely similarity with a language that they already master, spread among EU citizens or a combination of the two. Once the simulation is completed, the model calculates the rate of disenfranchisement under various conditions, namely how many people are not fluent in any EU official language under three possible language regimes.

To replicate the environment of the European Union, the agents are provided with a set of characteristics collected from actual databases. In the following subsections I explain how the database that feeds the model is constructed. The final database draws from two different databases, the Special Eurobarometer 386 and the linguistic distance database compiled by Dyen et al. [10].

### The Special Eurobarometer 386 survey

A number of properties are assigned to agents using data from the “Special Eurobarometer 386: Europeans and their Languages” [13]. The Eurobarometer surveys are conducted periodically on behalf of the European Commission and investigate many issues throughout its member states. They focus particularly on the citizens’ perception and expectations towards the intervention of the European Union and the challenges that it faces. The topics covered by the survey are numerous, ranging from air quality, gender equality and democracy to sports, trade and climate change.^13^ In particular, the Special Eurobarometer 386, carried out in 2012, is the latest survey concerning languages (similar surveys were carried out in 2001, 2005 and 2006) and provides information about language skills and the attitude of EU citizens towards multilingualism and language services. It covers the then 27 member states (the $28{\mathrm{th}}$ member state, Croatia, joined the EU in 2013). The survey includes information collected from 26,751 EU citizens from different social and demographic groups, aged 15 or older and residing in a EU member state, with a view to making the results of the survey as representative of the whole EU population as possible.^14^ In addition to information about languages, the database includes general demographic information, such as age, sex, profession and education. The survey collects data from roughly 1000 respondents per member state, except Cyprus, Luxembourg and Malta, with only about 500 respondents each. In the interviews, respondents were asked about the languages they speak, their motivation to learn foreign languages, the difficulties they encountered, the situations in which they resort to foreign languages, the impact of translation in their life, and so on.

For the purposes of the model presented here, I use a section of this database. For the practical reasons explained in Sect. “The linguistic proximity matrix”, four countries had to be excluded from the analysis, namely Finland, Hungary, Estonia and Malta. In the model, each agent reproduces the linguistic, social and demographic profile of an actual respondent from the survey. In particular, agents have the following properties:

1. country of residence;
2. nationality(ies);
3. age;
4. age when they finished education;
5. mother tongue(s);
6. profession (one of eight categories);
7. living condition (countryside, small city or big city);
8. first, second and third foreign language (if any) and related level of fluency (from 1, basic, to 3, very good).

As the focus is on the impact of language acquisition on linguistic disenfranchisement given the language regime, I look exclusively at the competences of citizens in the official languages of the EU countries considered, disregarding all other languages they might now. As a consequence, agents that spoke none of these languages were also excluded, leaving a database of 21,890 observations.

### The linguistic proximity matrix

In addition to the ones mentioned above, agents have an additional property, whose value depends on their mother tongue(s). Based on their native language, each agent takes on a vector of values that defines the linguistic proximity between their native language and all official languages of the environment simulated. The notion of linguistic proximity (or, equivalently, that of linguistic distance) is certainly interesting, but it poses many issues, in that it is highly dependent on the method used to compute it. For the purposes of the model developed here, I use the concept of linguistic proximity with a view to having a proxy of the subjective perception of each individual when it comes to picking a language that is closer to her native language and that she could learn relatively faster.

The information about linguistic distances comes from a different database, adapted from Dyen et al. [10]. The linguistic distance between languages is estimated using the lexicostatistical method, first introduced by Swadesh [28]. The method works by and large as follows:

- in the first phase, one prepares a list of basic terms that exist in all the languages that one wants to compare and collects the related terms;
- in the second phase, one looks at the terms for the same meaning across languages and establishes whether they are *cognates*, i.e., they descend from the same root;^15^
- in the third phase, one goes on to compute the percentage of cognate words within the list considered across pairs of languages.^16^

It is often more intuitive to speak of *linguistic proximity* rather than linguistic distance. The value of the linguistic proximity index goes from 0 (no cognate words in the list considered) to 1 (all words considered are cognate). Intuitively, the higher the value of the index, the closer the languages under study. For example, Swadesh [28] finds that English and German are connected by 57.8% of the 200 words that he considered (or, equivalently, have a proximity index of 0.578), while French and English are connected by 23.6% of the words (or have a proximity index of 0.236).

For the purposes of the model, it was necessary to create a 20 by 20 matrix that would include the pairwise linguistic proximity indexes for the Indo-European EU official languages. This choice stems from the fact that the original database by Dyen, Kruskal, and Black [10] only includes Indo-European languages. Therefore, the linguistic proximity index for some EU official languages, namely, Hungarian, Finnish, Estonian and Maltese, was not available. Therefore, these languages were excluded from the model. As a consequence, not to skew the results, Hungary, Finland, Estonia and Malta were also excluded from the database. The linguistic proximity indexes are reported in Table 2.

**Table 2:** Language proximity indexes between 20 Indo-European EU official languages (Dyen et al. [10])

	Irish	Romanian	Italian	French	Spanish	Portuguese	German	Dutch	Swedish	Danish
Irish	1	0.163	0.2	0.188	0.195	0.183	0.194	0.18	0.186	0.183
Romanian	0.163	1	0.66	0.579	0.594	0.629	0.249	0.254	0.239	0.237
Italian	0.2	0.66	1	0.803	0.788	0.773	0.265	0.26	0.259	0.263
French	0.188	0.579	0.803	1	0.734	0.709	0.244	0.244	0.244	0.241
Spanish	0.195	0.594	0.788	0.734	1	0.874	0.253	0.258	0.253	0.25
Portuguese	0.183	0.629	0.773	0.709	0.874	1	0.247	0.253	0.258	0.25
German	0.194	0.249	0.265	0.244	0.253	0.247	1	0.838	0.695	0.707
Dutch	0.18	0.254	0.26	0.244	0.258	0.253	0.838	1	0.692	0.663
Swedish	0.186	0.239	0.259	0.244	0.253	0.258	0.695	0.692	1	0.874
Danish	0.183	0.237	0.263	0.241	0.25	0.25	0.707	0.663	0.874	1
English	0.183	0.227	0.247	0.236	0.24	0.24	0.578	0.608	0.589	0.593
Lithuanian	0.196	0.203	0.242	0.221	0.23	0.215	0.224	0.214	0.218	0.222
Latvian	0.176	0.179	0.218	0.207	0.206	0.196	0.2	0.195	0.209	0.203
Slovenian	0.191	0.21	0.24	0.218	0.228	0.219	0.267	0.246	0.253	0.267
Czech	0.212	0.223	0.247	0.231	0.24	0.236	0.259	0.244	0.25	0.254
Slovakian	0.205	0.227	0.251	0.235	0.244	0.24	0.258	0.247	0.259	0.268
Polish	0.2	0.216	0.236	0.219	0.228	0.224	0.246	0.231	0.237	0.251
Bulgarian	0.182	0.202	0.231	0.209	0.218	0.219	0.231	0.221	0.236	0.24
Croatian	0.204	0.222	0.245	0.228	0.232	0.234	0.236	0.221	0.237	0.251
Greek	0.141	0.157	0.178	0.157	0.167	0.167	0.188	0.188	0.184	0.183

	English	Lithuanian	Latvian	Slovenian	Czech	Slovakian	Polish	Bulgarian	Croatian	Greek
Irish	0.183	0.196	0.176	0.191	0.212	0.205	0.2	0.182	0.204	0.141
Romanian	0.227	0.203	0.179	0.21	0.223	0.227	0.216	0.202	0.222	0.157
Italian	0.247	0.242	0.218	0.24	0.247	0.251	0.236	0.231	0.245	0.178
French	0.236	0.221	0.207	0.218	0.231	0.235	0.219	0.209	0.228	0.157
Spanish	0.24	0.23	0.206	0.228	0.24	0.244	0.228	0.218	0.232	0.167
Portuguese	0.24	0.215	0.196	0.219	0.236	0.24	0.224	0.219	0.234	0.167
German	0.578	0.224	0.2	0.267	0.259	0.258	0.246	0.231	0.236	0.188
Dutch	0.608	0.214	0.195	0.246	0.244	0.247	0.231	0.221	0.221	0.188
Swedish	0.589	0.218	0.209	0.253	0.25	0.259	0.237	0.236	0.237	0.184
Danish	0.593	0.222	0.203	0.267	0.254	0.268	0.251	0.24	0.251	0.183
English	1	0.216	0.197	0.249	0.241	0.25	0.239	0.228	0.234	0.162
Lithuanian	0.216	1	0.613	0.338	0.376	0.395	0.361	0.342	0.357	0.172
Latvian	0.197	0.613	1	0.323	0.333	0.357	0.332	0.306	0.337	0.152
Slovenian	0.249	0.338	0.323	1	0.663	0.694	0.633	0.615	0.684	0.179
Czech	0.241	0.376	0.333	0.663	1	0.914	0.766	0.689	0.719	0.164
Slovakian	0.25	0.395	0.357	0.694	0.914	1	0.778	0.685	0.732	0.168
Polish	0.239	0.361	0.332	0.633	0.766	0.778	1	0.631	0.68	0.163
Bulgarian	0.228	0.342	0.306	0.615	0.689	0.685	0.631	1	0.709	0.189
Croatian	0.234	0.357	0.337	0.684	0.719	0.732	0.68	0.709	1	0.179
Greek	0.162	0.172	0.152	0.179	0.164	0.168	0.163	0.189	0.179	1

### Simulating language learning

In the setup phase of the simulation, agents are created and residence is assigned proportionally to the actual distribution throughout the EU. As residence is more or less uniformly distributed in the original database (roughly 1000 observations per country), the model samples randomly out of it, to replicate the actual distribution of residents by country.^17^ All other properties are assigned by randomly selecting a respondent from the database and assigning her properties to an agent with the same residence, allowing for repetitions. When the simulation is launched, agents are asked to make a decision about learning a language. In the simplest case, an agent does not speak any EU language (other than her own). The agent is then asked, with a certain probability, to start learning a new EU language (I will discuss later how the agent selects the language to learn). In case the agent already knows one or more foreign languages, she is asked to look at her level of fluency in them (which, as said, goes from 1 to 3). If she speaks a foreign language at a level of fluency lower than 3, she is asked, with a certain probability, to go on learning it until she is proficient in it (i.e., she reaches level 3).^18^ If the agent’s foreign languages are all at level 3, she picks a new one with a certain probability, based on the rules explained below. This process goes on as a long as an agent knows less than three foreign languages at level 3.^19^

A property that does not belong to any agent but to the environment is the language regime. I consider three types of scenarios, that is, monolingual (monarchic), trilingual and hexalingual (oligarchic with, respectively, $n=3$ and $n=6$).^20^

**Fig. 1:**
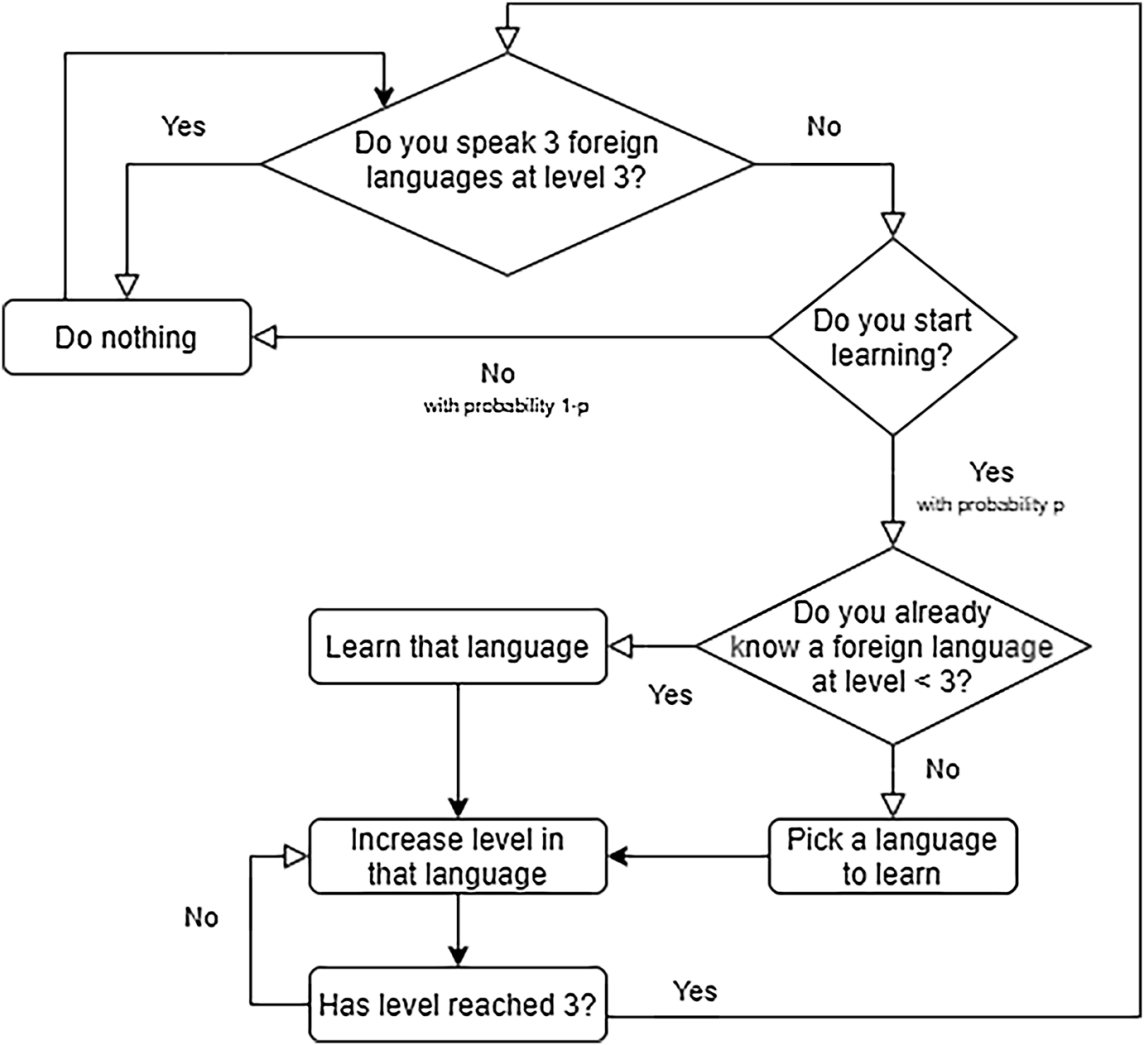
Flowchart of agents’ language selection strategy and learning behavior

In the first iteration, agents are asked to start learning a language with a certain probability *p*. Once they start learning a language, they keep learning until they become proficient in it. At every time step, an agent learns the language she has picked, that is, her level of fluency in that language increases. The speed at which she acquires skills in that language (that is, the speed at which her level in that language increases from 0 to 3) depends directly on the proximity between the language that she is trying to learn and the closest language among her native language(s) and the foreign language(s) in which she is fully proficient. The shorter the distance between the two languages, the fewer the time steps required by the agent to reach proficiency in the new language. When the agent has reached proficiency, she switches her status to “not learning” and the process starts again, that is, she will not be learning a new language, unless, again with probability *p*, she is not asked to do so. Every agent is programmed to be able to learn up to three foreign languages in total, including the ones that she already knows from the start. The behavior of agents is summarized in Fig. 1.

The languages are learned in succession and not in parallel. This is due to two reasons: first, I find that it is more reasonable for an individual to focus on the acquisition of one language at a time; second, as agents can base the choice of the language(s) to learn on their skills in all the languages in which they are proficient (native or non-native), becoming proficient in a foreign language can influence the pattern of future choices.

As has been explained before, if an agent already knows one or more foreign languages at a less-than-proficient level, it is assumed that she is currently learning it and will keep doing so, starting from the one that is closer to proficiency. When asked to pick a new language to learn, agents can then use one of three strategies, which is selected before the simulation is launched and applies to all agents:

1. they can pick the language that has the highest number of native (L1) and non-native (L2) speakers;
2. they can pick the language that is closest to (one of) their native language(s) or to any other language in which they are fluent, that is, the language they will learn in the shortest amount of time;
3. a combination of strategies 1 and 2, that is, they can pick a language close to their own and having a relatively high number of L1 and L2 speakers.

During the simulation, the model updates and keeps track of a number of values. It keeps track of the total number of L1 and L2 speakers of every language. This information is crucial and affects the model in two ways. At the micro-level, it affects individual agents’ decision about the language they should learn, if they are using strategies 1 or 3 in the list above. At the macro-level, this information is necessary for the system to establish the OWLs of the language regimes. Indeed, the three regimes consider, respectively, the one, three or six most spoken languages. After the simulation, I use the data generated to calculate the level of *relative* disenfranchisement, which is a direct consequence of the language regime.^21^ Finally, given that the model keeps the original information regarding the individual properties of the agents, it is possible to identify the socio-demographic categories most likely to be disenfranchised.

## Results and discussion

In this paper, I focus on the consequences of various approaches to language learning in terms of linguistic disenfranchisement. This can be interpreted as a harmonized education policy across EU member states to encourage citizens to acquire language skills on the basis of different rationales. I discuss two aspects of the results. First, I look at the changes in disenfranchisement rates across three different language regimes in three scenarios, one for each of the learning rules described in Sect. “Simulating language learning”. Then, I try to identify to categories more at risk of being disenfranchised, in terms of some of the properties mentioned in Sect. “The Special Eurobarometer 386 survey”.

In the simulations presented here, I assumed a probability of starting to learn a language equal to 1, to analyze the effect of the three different strategies under the general assumption that all actors are willing to learn. Indeed, a varying probability, although useful to simulate a scenarios in which not everybody decides to learn a language, would have mitigated the impact of the strategies. The initial population was randomly sampled from the database, as explained above, and then I ran three simulations, one for each strategy. Agents are allowed to learn during an arbitrary period of six iterations (which can be thought of as 6 years), during which the proficiency level in the language(s) that they learn increases according to the proximity between the language studied and the closest of the languages they speak fluently. The number of iterations might sound peculiar to some, as it is more common to see simulations models running hundreds or thousands of iterations in which few simple actions happen (see, for example, Civico [6]). Conversely, the simulation model presented here runs few iterations where a lot happens. This choice was based on the simple consideration that the model presented in this paper focuses on the acquisition of language skills by individuals. Obviously, learning a language, including one’s own native language, is a life-long process. As a consequence, I found that a time-span between 5 and 10 years was enough. Furthermore, the model should ideally be used to test policies to be implemented in the short term. As a matter of fact, it is not interesting to see whether, if the EU implemented a certain language regime today, people will no longer be disenfranchised, say, 25, 50, or 100 years from now. For situations such as the one discussed in this paper, it is only interesting to see whether a solution to the problem (in this case, disenfranchisement) can be worked out within a reasonable amount of time. I think that implementing language education orientations now and test whether they can solve an immediate communication problem in a quarter of a century would be not be of interest.

First, we can look at the numbers reported in Table 3, which displays the disenfranchisement rates associated with the three regimes across the various scenarios. The first column, “Initial situation”, reports the initial situation of disenfranchisement, before starting any learning process. As expected, the highest disenfranchisement rate is associated with the monolingual (English-only) regime, with almost 80% of the agents unable to speak English at a high level, either as a native or as a foreign language. The values associated with the two oligarchic regimes are clearly much lower, indicating that less people are left out when one of these regimes is adopted. Scenarios 1, 2 and 3 can be seen as three different directions in which the same initial situation evolves following the implementation of three different orientations in language education (i.e., the three different learning strategies explained earlier). Unsurprisingly, the disenfranchisement rates associated with the monolingual regime are still relatively high across all scenarios. However, although the differences are not particularly big, it is interesting to note that the monolingual regime is optimized under the first learning strategy, that is, learn the language with the highest number of fully proficient speakers (as L1 or L2), which is English.^22^ This is probably due to the fact that many agents already had a basic level of English and managed to reach proficiency during the simulation. For many other agents English was probably a close enough language to allow them to become fluent in it within the time allowed. However, there is still a sizeable amount of people who did not manage to become fluent in English in the time allowed for the simulation. This is mostly due to two facts. First, for many agents English is a distant language. Therefore, six iterations were not enough to allow them to reach fluency. Second, there were people who were already learning languages other than English, who might have managed to become fluent in those other languages. This last remark can be confirmed by the fact that the two oligarchic regimes under this same scenario are associated with much lower disenfranchisement rates.

**Table 3:** Disenfranchisement rates of different regimes across learning strategies (scenario 1, most widely spoken language; scenario 2, closest language; scenario 3, closest widely spoken language) and language regimes

	Initial situation (%)	Outcome after six iterations		
		Scenario 1 (%)	Scenario 2 (%)	Scenario 3 (%)
Monolingual	78.15	47.21	56.09	48.42
Trilingual	46.96	32.09	24.76	24.28
Hexalingual	19.04	9.60	6.09	3.03

Higher disenfranchisement rates imply that a bigger share of the population is not fluent in any official language

**Fig. 2:**
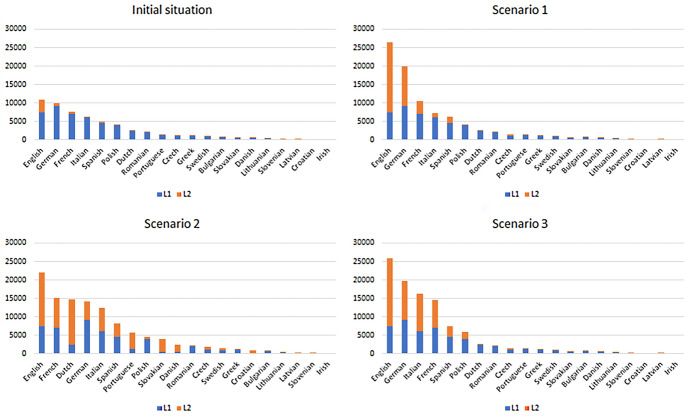
Distribution of L1 and L2 by total number of speakers across the different scenarios (scenario 1, most widely spoken language; scenario 2, closest language; scenario 3, closest widely spoken language)

The trilingual regime is almost equally optimized under scenarios 2 and 3, where people are allowed to learn the genealogically closer languages to any of the languages in which they are fluent (any language in scenario 2, one of the six most spoken languages in scenario 3). This strategy ensures that agents could learn new languages at a much faster pace, as they systematically pick the languages that would optimize their learning process. As could be expected, these two scenarios also grant a more uniform distribution of L2 skills, as opposed to scenario 1, in which L2 skills are more concentrated in the top three most spoken languages, as shown in Fig. 2, a bar chart reporting the various languages spoken in the simulated environment on the horizontal axis, in decreasing order of number of total speakers who speak that language as L1 or L2. The same arguments apply to the hexalingual regime, with the only difference that it is significantly better-performing under the conditions of scenario 3 in terms of disenfranchisement rate. This is most likely due to the fact that, under the conditions of scenario 2, agents too often pick languages to learn that would leave them disenfranchised. For example, a Bulgarian speaker would not pick Polish as her first choice, since there are other Slavic languages which are closer. However, Polish is the only Slavic language that is included in the hexalingual regime. Therefore, speakers of a Slavic languages are less likely to be disenfranchised if they pick a more widely spoken Slavic language, instead of simply the closest one. Another result that is worth noting is that Dutch ranks third in scenario 2. The reason of this result, which might seem surprising at first, is that there are many German and English speakers who, under the conditions of scenario 2, decide to learn Dutch, a genetically close language. As there are many L1-speakers of German and English in the simulated EU environment, it is not surprising that Dutch ranks so high.

I now move on to the second part of the analysis. One might argue that, if the strategies are adopted in the same way by all agents, the impact should be uniformly distributed among them. However, the interest of looking at the categories that are more susceptible of being disenfranchised stems from the fact that language skills in the database used to create the simulated population are not evenly distributed. Therefore, the uneven distribution of language skills might reverberate even after the implementation of the language learning policies, especially because it is assumed that agents can rely on their previously acquired language skills to learn new languages. For example, a French speaker would learn German at a relatively slow pace, because French and German are not very close. However, if this agent is fully proficient in English, she will be able to learn German at a faster pace, since she will be able to rely on her knowledge of English, which is closer to German. Besides, the languages most spoken across the countries considered are not equally close to all other languages. As a consequence, native speakers of certain languages could potentially learn them faster than others.

Several categories could be looked at. In this paper, I focus on a comparison of the hexalingual regime in scenarios 2 and 3 in terms of distribution of disenfranchised people across countries. I pointed out earlier that the hexalingual regime performs better in scenario 3, where agents learn the closest of the top six most spoken languages. However, if we look at the distribution of disenfranchised people (reported in Table 4), we can notice that, although the number of disenfranchised agents is lower in scenario 3, they tend to be much more concentrated in certain countries. In addition, under the conditions of scenario 2 some countries have more disenfranchised people than other, but the distribution is somewhat more even. This seems to suggest that, in terms of fair and equal treatment of all citizens, scenario 2 performs better than scenario 3.

**Table 4:** Distribution of disenfranchised people across countries (scenario 2,, closest language; scenario 3, closest widely spoken language)

Country	% of disenfranchised people by country	
	Scenario 2 (%)	Scenario 3 (%)
Belgium	2.07	3.96
Bulgaria	16.98	3.30
Cyprus	1.38	2.77
Czechia	21.29	3.69
Denmark	2.50	0.13
Germany	0.07	0.13
Greece	21.19	42.15
Ireland	0.07	0.13
Latvia	4.73	9.50
Lithuania	5.19	10.36
Netherlands	0.16	0.33
Poland	0.72	0.73
Portugal	3.65	6.99
Romania	5.26	10.49
Slovakia	7.10	1.72
Slovenia	2.83	2.90
Spain	0.43	–
Sweden	3.98	0.26
UK	0.43	0.46
Total	100.00	100.00

To better understand the value of computational models as a tool for policy making, it is also interesting to look at a breakdown of the disenfranchisement rate by social an demographic classes. There are many different classes that can be looked at, however, mainly for space reasons, I present only a breakdown of the disenfranchisement rate under scenario 1 in case of monolingual regime by profession, age group and living condition. Results are reported in Table 5.

**Table 5:** Breakdown of disenfranchised people by socio-demographic category (most widely spoken language + monolingual regime)

Profession	Disenfranchisement rate (%)	Age group	Disenfranchisement rate (%)	Living condition	Disenfranchisement rate (%)
Freelancer	42.97	15–24	35.13	Big city	39.54
Housemaker	**63.46**				
Manager	26.26	25–39	44.69	Countryside	**51.91**
Worker	**51.61**				
Retired	**49.33**	40–54	**50.08**	Small city	**48.06**
Student	27.65				
Unemployed	**61.34**	55+	**49.91**	Does not know	33.33
Other	43.59				
Overall	47.21	Overall	47.21	Overall	47.21

Average values of most widely spoken language and monolingual regime are given in bold

Each value reported in Table 5 indicates the percentage of people in that specific category who are disenfranchised. It is interesting to notice that, under the education orientation of scenario 1, if a monolingual regime was imposed, the most affected people would be housemakers, workers, or unemployed, aged 40 or older, living in a small city or in the countryside (values in bold in Table 5).

## Final remarks and future research

I conclude this paper by proposing some considerations about the usefulness of such an approach for policy making and about the specific model presented here. Furthermore, I address some of the limitations of the study and possibilities for future research.

There are two levels of considerations that can be made about the model presented in this paper. The first level concerns the use of computational models for policy making purposes. One of the objectives of this paper was to show that computational models are a valuable tool for policy makers. Indeed, they are very useful in the study of complex systems, that is, systems that are often better understood by looking at their constituent parts and the way they interact, rather than looking at the overall system itself. This is certainly the case of the model presented here. Instead of looking at the system of language learning from a strictly top–down perspective, this type of modeling allowed us to endow micro-level agents with a set of behavioral rules and then observe how their actions unfold and impact the macro-level system. Simulations make it possible to capture the heterogeneity of agents of complex systems. The same task is extremely complicated (if not utterly impossible) to do with other methodologies, such as equation-based models [11]. The agents of the model presented here are extremely heterogeneous under many aspects, not only demographic, but also in terms of their language skills. Furthermore, languages themselves have some properties that link them to one another, which influence the unfolding of the system and determine the outcome. As agents learn new languages, the relative weight of each language in terms of speakers varies, which in its turn affect the behavior of the agents. While this mechanism might seem trivial, it is really hard to capture its full complexity. Simulation models allow us precisely to do that, which is why I believe they represent a very valuable tool for policy makers. Obviously, it should be clear that simulation models do not represent an alternative to other more traditional research methods, but a complement. Indeed, their potential is fully exploited when they are used in combination with other qualitative and quantitative methods.

The second level of considerations concerns the results of the model itself. If full multilingualism should be abandoned by the EU institutions, policy makers should keep in mind that some parts of the population will inevitably be disenfranchised. However, disenfranchisement can be contained by an appropriate combination of language services and specific education orientations. A hexalingual regime, for example, seems to provide a lower disenfranchisement rate, which can be further decreased if the population adopts a language learning strategy that focuses on widely spoken languages that are genetically closer to their languages. In addition to this, it is important that policy makers take into consideration the fact that some parts of the population are more at risk of being negatively affected if the full multilingual regime should be abandoned. Therefore, regardless of the specific type of measure implemented, policy makers should make sure to include some form of compensation or safeguard for these classes.

This much being said, I want to point out that policy making is an eminently political process. Therefore, a discussion on whether a specific regime should be preferred or whether a fair and equal treatment of citizens should be prioritized is well beyond the scope of this paper and left for future research.

As concerns the limitations of the model presented in this paper, one of the most important things to point out is that it is strictly focused on understanding one aspect of language acquisition, that is, the consequences of linguistic distance and language learning in terms of linguistic disenfranchisement. Obviously, there are many other factors that come into play in the process of language acquisition, such as daily exposition to a given language in one’s daily life. An interesting development of the model presented here could be the inclusion of these other factors, with a view to studying their interaction. For example, one might be interested in seeing whether and to what extent exposition to a certain language through the media can compensate for the distance between languages.

For practical reasons, I have only taken a selection of EU official languages into consideration to calculate disenfranchisement indexes. However, the linguistic landscape of the EU has significantly changed over the past few years as a consequence of migration from the outside, and it is likely to keep on changing in the coming years. Migration can change the relative proportions of language speakers by either reinforcing one particular language group (say, Spanish speakers from South America) or creating allophone communities, i.e., groups of people speaking a non-EU language (for example, Chinese migrants). This picture becomes all the more intricate if we add to the equation all the regional and minority languages spoken throughout the EU. Besides, many people are able to speak more than one language in addition to their own, at different levels of proficiency. All in all, it can be challenging to sketch the linguistic landscape of the EU, let alone to predict its evolution. However, if EU institutions are to take linguistic disenfranchisement into consideration when drafting language policies, keeping track of changes in the linguistic landscape becomes crucial. With this objective, simulation models, as well as computational methods in general, are a good resource to have in the policy maker’s toolbox. For a more complete view of the EU linguistic landscape, an interesting extension to this model would be the inclusion of the remaining EU official languages and other non-EU languages. However, a non-trivial difficulty that would need to be tackled would be the modeling of linguistic distance encompassing also non-Indo-European languages among all of the language considered. If possible, it would be advisable to rely on more sophisticated metrics of language distance, in that, as said, the linguistic distance index has its flaws.

This study was started when the UK was still part of the European Union. As of today, it is still not clear what is going to be the future of English within the EU institutions. The relative weight of English in terms of native speakers was drastically reduced after Brexit, and this could have repercussions not only on the communication practices among EU employees, but also on education policies across various member states. Therefore, it would be interesting to repeat this study in a few years, leaving some time for the linguistic repercussions of Brexit to manifest.^23^

Finally, another limitation concerns the choice of the probability with which agents decide to learn a language, which is set equal to one. The reason why I structured the model in such a way is that simulation models are often used to test scenarios which would be impossible to implement in real life, for various reasons, such as ethical concerns. Nevertheless, these models are useful to gauge the impact of certain policy measures to get an idea of their consequences before actually implementing them. In the specific case of the model presented here, I simulated the extreme case in which every agent decides to learn a language to estimate the maximum potential of such policy measures. However, it goes without saying that policy makers should always keep in mind that actual measures, which needs to be informed not only by simulation models but also by other types of considerations, need to be designed so that they respect, among other things, ethical, legal and economic constraints.

## Data Availability

The complete code of the model and the database used to run the simulations are available at https://www.comses.net/codebase-release/554ba57d-f370-4453-99f4-9b9f800c193d/.

## Footnotes

1 It should be noted, however, that this is certainly true in theory, but not always in practice. For example, it was noted that the communications drafted by the EU to address the Covid-19 pandemics emergency were only available in English, which, at the time when these documents were published, was the native language of only 2 percent of the EU population. For more on this, see https://www.corriere.it/opinioni/20_febbraio_26/anche-emergenze-a993f9c2-58c6-11ea-8e3a-a0c8564bd6c7.shtml.

2 For an in-depth review of models of language competition, see Boissonneault and Vogt [3].

3 See https://www.ethnologue.com/region/Europe.

4 For more on the EGIDS, see https://www.ethnologue.com/language-development.

5 For the complete text of the Treaty, see https://www.coe.int/en/web/conventions/full-list/-/conventions/rms/0900001680695175.

6 The authors define linguistic proximity as a value from 1 to 5 calculated from the number of common nodes in a linguistic classification tree between the closest official languages of pairs of countries.

7 In this regard, it is worth noting that Articles 3 and 4 of the Regulation distinguish between documents to be sent to a specific member state or individual falling under the jurisdiction of a member state, which shall be drafted in the official language of the member state, and documents of general application, which shall be drafted in all official languages.

8 See http://europa.eu/rapid/press-release_MEMO-13-825_en.htm.

9 Since February $1{\mathrm{st}}$, the number of member states went down to 27, but the number of official languages is still 24. For the purposes of the model presented here, the United Kingdom is still taken into consideration.

10 For the full text of the Gaelic Language (Scotland) Act 2005, see http://www.legislation.gov.uk/asp/2005/7.

11 The numbers are updated to account for the addition of Croatian in the set of OWLs.

12 For an in-depth discussion of linguistic distance and how it can be computed, see Dyen et al. [10].

13 For a list of the topics covered by the Eurobarometer surveys, see https://ec.europa.eu/COMMFrontOffice/publicopinion/index.cfm

14 The sampling procedure is clearly detailed in the appendix to the Eurobarometer 386. In short, the data were collected through a multistage random sampling by drawing from each country a number of sub-units with probability proportional to population size and density. These random sub-units were then once again sampled at random down to household level. To ensure the right coverage of the EU territory, sub-units were collected from each “administrative regional unit” according to the EUROSTAT NUTS II classification. Finally, interviews were conducted in person in the respondent’s home and in the appropriate national language.

15 It should be noted that two terms in two different languages are considered cognates if and only if they are descended from the same ancestors, and not if a language simply borrowed the term from the other.

16 Another valid option to calculate linguistic distance that is worth mentioning here is provided by Wichmann et al. [30]. These distance indexes are also calculated on the basis of the word list provided by Swadesh, but using a modified version of the so-called Levenshtein distance (LD).The Levenshtein distance between two words is defined as the minimum number of edits (insertions, deletions or substitutions of characters) that one needs to perform to turn one word into the other. The authors focused on phonological similarity and calculate the pairwise distance between languages as the average LD of pairs of words having the same meaning and they then normalize it by the average LD of pairs of words having different meanings, to account for similarity due to chance.

17 In the simulation performed, I sample 50000 agents (with repetitions) with the following distribution: Austria, 915; Belgium, 1177; Bulgaria, 707; Cyprus, 123; Czech Republic, 1088; Denmark, 589; France, 6633; Germany, 8513; Greece, 1059; Ireland, 502; Italy, 6144; Latvia, 191; Lithuania, 276; Luxembourg, 64; Netherlands, 1741; Poland, 3846; Portugal, 1037; Romania, 1955; Slovakia, 554; Slovenia, 211; Spain, 4751; Sweden, 1026; United Kingdom, 6898.

18 In case the agent speaks more than one foreign language at the same level of fluency, she picks one of them based on the rules explained below.

19 I am aware that this is a relatively extreme choice, given that not everyone is willing (or simply has the chance) to learn three foreign languages. However, I can make two considerations to justify this choice. First, this number is certainly a limit, but only a very small minority of agents get to learn three foreign languages in the time allowed for the simulation. Therefore, it should rather be seen as a possibility to learn more languages, should an agent be able to do so. Second, having a high cap to the number of languages can be interpreted as a context in which the acquisition of language skills is highly encouraged, which is the scenario that I am interested in exploring.

20 In the simulated database, the six most spoken languages as native or foreign language (at a proficient level) are, in descending order, English, German, French, Italian, Spanish and Polish. These are the languages taken into consideration for the three language regimes.

21 As I reminder from Sect. “Linguistic disenfranchisement”, the *relative* disenfranchisement rate considers disenfranchised all people who are not native or proficient users of an official language. Conversely, the *absolute* disenfranchisement rate considers disenfranchised all individuals who have absolutely no knowledge of an official language.

22 Obviously, if an agent is already fluent in English, she will start learning the second or third most spoken language.

23 For an interesting discussion on this matter, as well as on the dominant position of English in the EU, see https://www.publico.pt/2016/07/01/mundo/opiniao/as-linguas-na-europa-o-que-mudara-com-o-brexit-1736870.
